# The correlation between dietary fat quality indices and lipid profile with Atherogenic index of plasma in obese and non-obese volunteers: a cross-sectional descriptive-analytic case-control study

**DOI:** 10.1186/s12944-020-01387-4

**Published:** 2020-09-26

**Authors:** Maryam Sadat Moussavi Javardi, Zahra Madani, Ariyo Movahedi, Majid Karandish, Behnood Abbasi

**Affiliations:** 1 Department of Nutrition, Science and Research Branch, Islamic Azad University, Tehran, Iran; 2 Nutrition and Metabolic Diseases Research Center, Ahvaz Jundishapur University of Medical Sciences, Ahvaz, Iran

**Keywords:** Fat quality, Atherogenic index of plasma, Lipid profile, Obesity, Overweight, Atherogenicity, Thrombogenicity, Cholesterol-saturated fat index

## Abstract

**Background and aim:**

Abnormalities in lipid metabolism are commonly observed in patients who were obese. Alongside dyslipidemia, one of the markers in predicting the risk of cardiovascular disease is the Atherogenic Index of Plasma (AIP), which is related to dietary intake. Healthy fat quality indices might affect on AIP. The purpose of this study is to find the possible relationship between dietary fat quality, and AIP and comparison of these indices among obese and non-obese volunteers.

**Methods:**

This study was a cross-sectional descriptive-analytic case-control study with 157 normal and overweight and obese volunteers (*n* = 71 normal, Age: 38.90 ± 10.976 vs *n* = 86 overweight/obese, Age: 38.60 ± 9.394) in the age range of 18–65 years. Food intake was measured using FFQ, anthropometric indices (weight, height, body mass index and waist to hip ratio), body composition (visceral fat level, total body water, body fat mass), and lipid profile were measured.

**Results:**

Based on the present results, comparable biochemical parameters including TC (*P =* 0.580), TG (*P =* 0.362), LDL (*P =* 0.687) and HDL (*P =* 0.151) among overweight/obese volunteers as compared to normal ones were noticed. Effects of dietary fat quality, including Atherogenicity (AI) and Thrombogenicity (TI) hypo/hypercholesterolemic ratio (h/H), the Cholesterol-Saturated Fat Index (CSI) showed significantly higher AI (*P* = 0.012) in the overweight/obese group as compared to the normal group. Whereas, h/H (*P =* 0.034) and ω-6/ω-3 ratio (*P =* 0.004) were significantly higher in normal-weight volunteers. There was a positive correlation between AI, TI, CSI, SFA, MUFA, PUFA and ω-6/ω-3 ratio with AIP and negative correlation between h/H with AIP in both groups. Despite the significances of these correlations no strong relation was observed by doing multiple regression among normal and overweight/obese groups (*R*^2^ = 0.210, *R*^2^ = 0.387).

**Conclusions:**

In summary, the present work proposes a direct relationship between dietary fat quality, increased BMI, and lipid abnormalities with AIP. Nevertheless, further large-scale studies are required to sustain a clear conclusion in this wish.

## Background

Nowadays, non-communicable diseases (NCDs) are the most important worldwide health issue. Among NCDs obesity and hyperlipidemia are two main metabolic disorders that increase the risk of developing cardiovascular disease [1]. Obesity is classified as a category of chronic diseases [2, 3] and it is recognized to be an inflammatory state with increased adipose tissue and reduced levels of adiponectin, which limits its ability to suppress inflammatory processes and perpetuates the inflammatory condition [4–6]. Also, perivascular adipose tissue seems to impair local inflammation and endothelial function, particularly in obese people. Obesity due to increased intravascular inflammation and interstitial arterial thickness and decreased arterial lumen diameter reduces vascular elasticity, which eventually leads to hypertension [7]. Arterial stiffness increases systolic blood pressure (SBP) while reducing diastolic blood pressure (DBP). These consequences along with elevated pulse pressure raise the strain on the left ventricle, leading to increased risk of myocardial infarction and other coronary heart diseases (CHD) [8–10]. The global worldwide rate of CVD, which is a consequence of pandemic obesity, is expected to reach 23.6 million by 2030 [11]. Granting to the latest data published by the World Health Organization in 2018, a rapidly increasing rate of obesity was seen worldwide, and, more than 2 billion adults aged 18 years and older were overweight. Of these, over 650 million adults were obese (WHO, 2018), and the United States at the forefront, because approximately 35% of men and 40% of obese women are defined as having a body mass index (BMI) > 30 kg/m^2^ [12, 13].

On the other hand, abnormalities in lipid metabolism were noted commonly in patients who were obese. Substantial indicated that high BMI is directly or indirectly linked to high total cholesterol (TC), low-density lipoprotein cholesterol (LDL-C), and triglycerides (TG) and an inverse relationship with high-density lipoprotein cholesterol (HDL-C). Strong scientific evidence indicates that there is a solid association between BMI and lipoprotein levels, in particular, high levels of LDL-C and also a low level of HDL-C, which was proposed as a potential risk factor for CVD in obese people [14]. Therefore the LDL-C/HDL-C ratio is often calculated to estimate cardiovascular risk [15]. A more sensitive and specific index of cardiovascular risk than total cholesterol as a TC/HDL ratio, which higher than 5.5 indicates the moderate atherogenic risk [16]. Alongside dyslipidemia, one of the strongest markers in predicting the risk of CVD is the atherogenic index of plasma (AIP). Atherogenic index of plasma (AIP) is a novel index [17], which has been used to measure blood lipid levels and is usually used as an optimal indicator of dyslipidemia and associated disorders like cardiovascular diseases [18–20].

The number of nutrients and intake of their type, along with the calorie imbalance are the most important factors in weight gain and obesity. It has shown that not only each of the macronutrients such as fat, carbohydrates, and protein has different effects on weight changes [21], but also it has shown that the type and quality of the foods have different effects [22]. For instance, during the last decades, previous studies proposed some indices of dietary fat quality, including the atherogenicity index (AI) and thrombogenicity index (TI), and the ratio between hypo -hypercholesterolemic fatty acids (h/H) in the diet which might have effects on CVD and other NCD Risks [23, 24]. In 1996, Mitchell et al. have proposed another index of dietary fat quality which named Cholesterol-Saturated Fat Index (CSI) [25] and a few years later, in 2002, Simopoulos et al., proposed the importance of the ratio of omega-6/omega-3 essential fatty acids [26].

Despite having all these indices, no studies have investigated the quality of fat intake, and their possible association with AIP changes. Hence, this study aimed to find out the possible relationship between these dietary factors and AIP and compare it among overweight/obese and non-obese volunteers.

## Methods

### Sample collection and preparation

This study was a cross-sectional descriptive-analytic case-control study, which has been done during May 2019 till September 2019 in Tehran. Based on a sample size formula which has been calculated by using PASS 15.0 (Power Analysis and Sample Size software, NCSS LLC., Utah, USA), 128 subjects were required totally. In this study, stratified sampling method was used based on Age range, BMI, pregnancy, medical drug use. After choosing eligible samples from the main data bank, they were invited to be a volunteer in this study. Volunteers who were willing to cooperate, BMI > 18.5 and the age range 18–65 years, and were not pregnant or under anti dyslipidemia drugs were included in this study. The volunteers were normal weight (18.5 ≤ BMI < 25 Kg/m^2^) and overweight or obese (BMI ≥ 25 Kg/m^2^) adults who were randomly selected from the students and staffs of Science and Research Branch of Islamic Azad University (SRBIAU) of Tehran using stratified sampling method [27]. Overall, 71 normal weight and 86 overweight/obese volunteers were completed study missions. All the basic required information, including BMI and the latest blood test, were available in the University Electronic Health Clinic Database. Based on the registered documents in the SRBIAU Health Clinic Database, to collect the blood samples of volunteers, they have fasted overnight for 12 h and serum blood has been taken and centrifuged using Boeco U-320 Pathology Laboratory Centrifuge (Boeco, Hamburg, Germany). The enzymatic colorimetric methods have been used to determine the serum concentrations of the lipid profile using a Cobas C-311 analyzer (Roche, Meylan, France). Blood pressure was measured by Automatic, Noninvasive Blood Pressure Measurement BPBIO 320S (InBody BPBIO320, Eonju-ro, Gangam-gu, South Korea) [28].

### Study implementation

This study was approved by the ethical Iran National Committee for Ethics in Biomedical Research under code IR.IAU.SRB.REC.1396.67. All the eligible volunteers were informed about the details of the study and their rights to sign a written consent.

All the basic characteristics including age and sex were obtained through face-to-face interviews by valid questionnaires. Anthropometric indices, including weight, BMI, WHR, and body composition (visceral fat level, total body water, body fat mass) were measured using the InBody Model 270 bioelectric impedance analyzer (InBody Co. Ltd., Eonju-ro, Gangam-gu, South Korea) and height was measured using digital freestanding Stadiometer BSM-170 (InBody Co. Ltd., Eonju-ro, Gangam-gu,South Korea). Dietary data were collected using a validated Iranian semi-quantitative FFQ with 147 food items [29].

### Fat quality indices

To estimate of dietary fat quality indices derived from previous studies calculated using empirical equations are:

#### Atherogenic index (AI) formula

Indicates a correlation between the total saturated and unsaturated fatty acids. Is the sum of C12:0 = Lauric acid, C14:0 = Myristic acid, C16:0 = Palmitic acid, ∑MUFA = sum of monounsaturated fatty acids, ∑ɷ-6 = sum of omega-6 polyunsaturated fatty acids, ∑ɷ-3 = sum of omega-3 polyunsaturated fatty acids [23].

$$ (AI)=\frac{\left[\left(C12:0+\left(4\ x\ C14:0\right)+C16:0\right)\right]}{\left(\sum MUFA+\sum \varpi -6+\sum \varpi -3\right)} $$

#### Thrombogenic index (TI) formula

MUFA and n-6 PUFA are less anti-atherogenic than n-3 PUFA. C14:0 = Myristic acid, C16:0 = palmitic acid, C18:0 = stearic acid, ∑Sn-6 = total omega-6 fatty acids, ∑Sn-3 = total omega-3 fatty acids, and ∑MUFA = sum of monounsaturated fatty acids [23].

$$ \left(\mathrm{TI}\right)\kern0.5em =\kern0.5em \frac{\left(C14:0\kern0.5em +\kern0.5em C16:0\kern0.5em +\kern0.5em C18:0\right)}{\Big[\left(0.5\ \mathrm{x}\sum \mathrm{MUFA}\right)+\left(0.5\ \mathrm{x}\sum \varpi -6+\left(3\ \mathrm{x}\sum \varpi -3\right)+\left(\sum \varpi -3/\sum \varpi -6\right)\right]} $$

#### Ratio of hypo and hypercholesterolemia (h/H)

C18:1 n-9 = Oleic acid, C18:2 n-6 = Linoleic acid, C20:4 n-6 = Arachidonic acid, C18:3 n-3 = Alpha-linolenic acid, C20:5 n-3 = Eicosapentaenoic acid, C22:5 n-3 = Docosapentaenoic acid, C22:6 n-3 = Docosahexaenoic acid, C14:0 = Myristic acid, C16:0 = Palmitic acid [24].

$$ \left(\mathrm{h}/\mathrm{H}\right)=\frac{\left(C18:1\varpi -9+C18:3\varpi -6+C18:3\varpi -3+C20:5\varpi -3+C22:6\upomega -3\right)\ }{\left(C14:0+C16:0\right)} $$

#### Cholesterol/saturated fat index (CSI)

The Cholesterol-Saturated Fat Index (CSI) has potential as a dietary self-monitoring tool and enables patients to monitor their progress toward a cholesterol-lowering [25].

$$ \left(\mathrm{CSI}=\right)\frac{Cholesterol}{Saturated\  fat} $$

#### Atherogenic index of plasma (AIP) formula

To evaluate the logarithm of the ratio of plasma concentration of triglycerides to HDL-C [17].

$$ AIP\kern0.5em =\kern0.5em Log\kern0.5em \left[\frac{(TG)}{\left( HDL-C\right)}\right] $$

### Statistical analysis

Kolmogorov-Smirnov test as well as D’Agostino-Pearson omnibus test was used to find out the normality of the tested variables [30]. The Student’s t-test was used to compare the mean of quantitative (for parametric distributions) and Mann–Whitney U test (for nonparametric distributions) was used to compare the median of outcomes between the two groups. The correlation model was used to compare the main variables of the study in case of a need for control over the main variables of the study. Also, a multiple regression test was used to predict the effects of variables on AIP. IBM SPSS Statistics for Windows version 25 (IBM Corp., Armonk, N.Y., USA) was used for all analyses, a *p*-value of 0.05 or less was considered to be significant with a confidence interval of 95%.

## Results

In the present study, the relationship between dietary fat quality indices with an AIP in overweight/obese and non-obese volunteers, with a mean age of 38.73 ± 9.65 years in both groups were evaluated. One hundred eighty adults, 90 overweight/obese, and 90 normal weight were enrolled based on inclusion and exclusion criteria. Of these, 23 subjects were excluded because of incomplete questionnaires (more than half of the items were not completed) and some had over/under-reporting FFQ. Finally, the study was done on 71 normal weight and 86 overweight and obese volunteers.

As shown in Table 1, all anthropometric indices except height were significantly higher among the overweight and obese group (*P <* 0.001). Moreover fat mass was significantly higher in the overweight/obese group as compared to the normal ones (*P <* 0.001). Both bone mass and total body water showed comparable differences. Biochemical results showed no significant difference for TC (*P =* 0.580), TG (*P =* 0.362), LDL (*P =* 0.687), and HDL (*P =* 0.151) and significant difference for AIP (*P =* 0.014), between overweight/obese subjects and normal subjects. SBP and DBP were observed significantly higher in the overweight/obese group as compared to the normal group (*P <* 0.001). Also, a comparable pulse rate was found in the overweight/obese group (*P =* 0.327).

**Table 1:** Descriptive characteristics of study participant

	Normal weight (71)				Overweight/Obese (86)				*P*-Value
	***Mean ± SD***	Median	Mode	Range	***Mean ± SD***	Median	Mode	Range	
Sex (M/F)	80.3% / 19.7%	–	–	–	81.6% / 18.4%	–	–	–	0.731
Age (year) ^a^	38.90 ± 10.976	39	30	18–40	38.60 ± 9.394	38	28	19–47	0.854
*Anthropometric characteristics*									
Weight (Kg) ^a^	73.45 ± 10.66	74.1	69.0	47–85	90.05 ± 13.22	88.4	86.5	65–110	0.0001**
Height (Cm) ^a^	172.54 ± 7.58	171	174.5	157–188	172.20 ± 8.33	169.5	173	156–182	0.789
BMI (Kg/m^2^) ^a^	24.57 ± 2.32	23.2	22.8	19.2–25	30.28 ± 3.16	31.3	30.08	25.4–36.6	0.0001**
WHR(Cm)^b^	0.90 ± 0.04	0.87	0.89	0.83–0.92	0.95 ± 0.06	0.99	0.98	0.92–1.03	0.0001**
Skeletal Muscle Mass (Kg) ^a^	33.07 ± 3.12	34.01	32.78	28.23–37.41	30.93 ± 4.86	29.32	31.17	24.02–39.81	0.0001**
Fat Mass(Kg) ^a^	26.74 ± 5.06	26.12	27.31	20.23–33.18	37.54 ± 4.94	35.33	36.46	32.23–42.93	0.0001**
Bone Mass (Kg) ^a^	4.03 ± 0.79	4.13	4.27	3.03–4.97	4.22 ± 0.66	4.32	4.37	3.43–5.07	0.821
Total Body Water	42.79 ± 8.17	43.90	41.03	31.23–53.45	40.51 ± 7.97	41.88	40.71	30.36–50.14	0.119
*Biochemical parameters*									
TG (mg/dl) ^b^	152.52 ± 85.38	144	139	66–237	164.99 ± 84.67	159	151	82–263	0.362
TC (mg/dl) ^a^	172.17 ± 35.30	169	166	130–208	175.06 ± 29.90	177	179	143–207	0.580
LDL (mg/dl) ^a^	98.44 ± 29.71	96	99	65–128	100.22 ± 25.75	101	102	73–132	0.687
HDL (mg/dl) ^a^	44.51 ± 8.41	46	45	35–54	42.40 ± 9.67	42	39	30–50	0.151
AIP^b^	0.170 ± 0.07	0.161	0.166	0.16–0.18	0.214 ± 0.111	0.221	0.232	0.115–0.326	0.014*
*Blood pressure, mean ± SD*									
SBP (mmHg) ^a^	121.17 ± 13.03	125	115	105–135	131.02 ± 15.18	140	135	115–150	0.0001**
DBP (mmHg) ^a^	76.90 ± 11.13	75	80	65–90	84.21 ± 11.43	90	85	75–10	0.0001**
Pulse (bpm) ^a^	84.55 ± 13.20	83	82	70–99	86.63 ± 13.19	85	83	73–102	0.327

^a^ Variables were compared using t independent test; ^b^ variables were compared using Mann–Whitney U test. Percentages are compared using Chi-square. * Stands for *P <* 0.05. ** Stands for *P <* 0.01. Comparison between in two groups of normal and overweight or obese; *P <* 0.05 was considered to be significant

According to the findings of Table 2, the results showed that AI (*P =* 0.012) was significantly higher in the overweight/obese group, whereas, h/H (*P =* 0.034) and ω-6/ω-3 ratio (*P =* 0.004) were higher significantly in normal weight subject than overweight/obese subjects.

**Table 2:** The Comparison of fat quality intake in two groups of normal and overweight or obese

Variable	Normal weight (***n*** = 71)				Overweight/Obese (***n*** = 86)				***P***-Value
	Mean ± SD	Median	Mode	Range	Mean ± SD	Median	Mode	Range	
AI^a^	228.19 ± 28.32	226.3	203.04	197.02–260.21	461.48 ± 52.81	456.93	469.12	408.08–519.01	0.012*
TI^a^	471.15 ± 46.14	474.64	456.09	436.78–522.66	484.45 ± 50.63	474.64	465.96	431.96–537.84	0.841
hH^a^	71.85 ± 3.78	69.21	70.43	66.86–75.64	49.94 ± 4.13	50.69	49.97	43.35–55.14	0.034*
CSI^a^	30.40 ± 0.45	30.03	30.64	29.88–31.22	31.00 ± 0.93	31.10	30.94	30.07–32.33	0.824
Total fat	90.30 ± 35.51	89.11	74.07	55.09–126.98	95.52 ± 42.73	96.78	97.01	52.49–137.24	0.412
∑SFA	19.55 ± 8.01	20.03	18.93	11.04–27.68	22.01 ± 10.40	21.54	21.82	11.76–32.47	0.066
*Loric acid (c12:0)*	0.40 ± 0.27	0.39	0.43	0.12–0.68	0.41 ± 0.27	0.40	0.45	0.13–0.71	0.715
*Myristic acid (14:0)*	1.43 ± 0.87	1.32	1.50	0.57–2.30	1.56 ± 0.88	1.62	2.02	0.67–2.48	0.325
*Palmitic acid (16:0)*	8.43 ± 3.64	8.41	9.93	4.80–12.02	9.34 ± 4.67	9.35	8.28	4.72–14.01	0.875
*Stearic acid (18:0)*	4.00 ± 1.78	4.14	3.94	2.21–5.78	4.42 ± 2.41	4.52	4.78	2.01–6.71	0.062
∑MUFA	23.99 ± 11.38	24.41	22.86	12.04–35.18	21.97 ± 8.61	22.11	22.29	13.34–30.57	0.207
*Oleic acid (18:1)*	15.55 ± 8.43	15.98	14.07	7.33–19.42	13.38 ± 6.10	13.47	11.55	7.28–19.46	0.812
*Palmitoleic acid (16:1)*	0.79 ± 0.63	0.72	0.70	0.17–1.43	0.69 ± 0.43	0.68	0.93	0.24–1.15	0.209
∑PUFA	19.25 ± 12.20	21.22	15.03	7.20–30.03	22.45 ± 12.72	21.57	19.31	8.95–33.94	0.920
*Linoleic acid (18:2n6)*	1.22 ± 1.34	1.25	0.73	0.14–2.57	1.45 ± 1.24	1.59	1.08	0.20–2.69	0.267
*C18.2.CLAs*	0.41 ± 0.72	0.31	0.36	0.29–1.15	0.23 ± 0.32	0.28	0.21	0.09–0.58	0.144
*Linolenic acid (18:3n6)*	0.12 ± 0.14	0.09	0.13	0.03–0.30	0.17 ± 0.13	0.11	0.16	0.05–0.33	0.177
*Linolenic acid (18:3n3)*	0.41 ± 0.27	0.45	0.38	0.12–0.70	0.52 ± 0.27	0.49	0.56	0.24–0.79	0.565
*Dihomo-linolenic acid (20:3n6)*	0.34 ± 1.45	0.31	0.26	0.10–1.88	0.42 ± 1.23	0.45	0.37	0.08–1.79	0.129
*Arachidonic acid (20:4n6)*	0.11 ± 0.10	0.09	0.07	0.01–0.23	0.18 ± 0.08	0.19	0.16	0.08–0.29	0.212
*Eicosapentaenoic acid (20:5n3) EPA*	0.04 ± 0.05	0.03	0.02	0.01–0.11	0.03 ± 0.04	0.02	0.02	0.01–0.08	0.615
*Docosahexaenoic acid (22:6n3) DHA*	0.13 ± 0.12	0.11	0.05	0.01–0.26	0.12 ± 0.11	0.10	0.08	0.01–0.25	0.779
ω-6/ω-3 ratio	4.27 ± 0.29	4.25	4.50	3.98–4.59	4.60 ± 0.27	4.61	4.52	4.32–4.89	0.004**
Cholesterol	224.36 ± 112.73	228.65	201.12	110.14–332.38	250.89 ± 174.34	254.08	247.36	87.12–413.97	0.250
Total trans	4.39 ± 10.81	3.21	3.89	3.13–15.29	5.58 ± 10.27	3.98	4.27	3.51–10.09	0.481

^a^ Variables were compared using t independent test, rest of the variables were compared using Mann–Whitney U test. * Stands for *P <* 0.05. ** Stands for *P <* 0.01. Abbreviation: *AI* Atherogenicity index; *TI* Thrombogenicity index; *H/H* Σhypocholesterolemic/Σ Hypercholesterolemic ratio; *CSI* Cholesterol-Saturated Fat Index; *ω-6/ω-3* Σ of Omega 6 series/Σ of Omega 3 series; *SFAs* saturated fatty acids; *MUFAs* monounsaturated fatty acids; *PUFAs* polyunsaturated fatty acids; *ω6* omega 6 fatty acid (Linoleic acid); *ω3* omega 3 fatty acid (Linolenic acid); *P <* 0.05 was considered to be significant

Comparable differences were found for SFAs, including Loric (*P =* 0.715), Palmitic (*P =* 0.875), Stearic (*P =* 0.062), and Myristic (*P =* 0.325) acids, total trans (*P =* 0.481), Cholesterol (*P =* 0.250) and MUFA (*P =* 0.207) among the overweight/obese group. The same result was observed for PUFA as well (*P =* 0.920).

As illustrated in Table 3, there was a positive correlation between BMI, AI, TI, CSI, SFA, MUFA, PUFA, and ω-6/ω-3 ratio with AIP and negative correlation between h/H with AIP in both groups. Significant correlations were observed between BMI (*P =* 0.045, *R* = 0.408), AI (*P =* 0.014, *R* = 0.859), h/H (*P =* 0.033, *R* = -0.596) and SFA (*P =* 0.043, *R* = 0.602) with AIP in overweight/obese group and were significant for the AI (*P =* 0.031, *R* = 0.701) and h/H (*P =* 0.023, *R* = − 0.710) in normal group. Despite this significance, multiple regression between these variables with AIP showed a weak relationship among the normal group (*R*^2^ = 0.210) and comparable one among the overweight/obese group (*R*^2^ = 0.387).

**Table 3:** The Correlation coefficient between BMI, dietary fat quality indices with AIP in normal weight and overweight or obese groups

Variable	Normal weight (***n*** = 71)			Overweight/Obese (***n*** = 86)		
	R	***P*** value	R^**2**^	R	***P*** value	R^**2**^
BMI	0.193	0.106	0.210	0.408	0.045*	0.387
AI	0.701	0.031*		0.859	0.014*	
TI	0.080	0.505		0.095	0.381	
CSI	0.085	0.481		0.024	0.117	
hH	-0.710	0.023*		−0.596	0.033*	
∑SFA	0.050	0.925		0.602	0.043*	
∑MUFA	0.041	0.416		0.015	0.403	
∑PUFA	0.005	0.960		0.098	0.581	
ω-6/ω-3 ratio	0.179	0.135		0.087	0.425	

* Stands for *P <* 0.05. R: Pearson’s correlation coefficient, Abbreviation: *AI* Atherogenicity index; *TI* Thrombogenicity index; *h/H* ΣHypocholesterolemic/ ΣHypercholesterolemic ratio; *CSI* Cholesterol-Saturated Fat Index; ω-6/ω-3 = Σ of Omega 6 series/Σ of Omega 3 series; *SFAs* saturated fatty acids; *MUFAs* monounsaturated fatty acids; *PUFAs* polyunsaturated fatty acids; *ω6* omega 6 fatty acid (Linoleic acid); *ω3* omega 3 fatty acid (Linolenic acid). Tests were anylysed using Pearson’s correlation and multiple regression test

As Table 4 presents, a significant correlation was observed between lipid profile and AIP in normal weight and overweight/obese groups. The positive correlation was significant between TG, TC, LDL, TC/HDL, LDL/HDL with AIP except for HDL that negative correlation was significant in both groups (*P <* 0.05, *R*^2^ = 0.889, *R*^2^ = 0.878, normal and overweight/obese group respectively).

**Table 4:** The Correlation coefficient between lipid profile and AIP in normal weight and overweight/obese groups

Variable	Normal weight (***n*** = 71)			Overweight/Obese (***n*** = 86)		
	R	***P*** value	R^**2**^	R	***P*** value	R^**2**^
TG	0.909**	0.0001	0.889	0.919**	0.0001	0.878
TC	0.481**	0.0001		0.302**	0.004	
LDL	0.318**	0.007		0.494**	0.008	
HDL	−0.632**	0.0001		−0.708**	0.0001	
TC/HDL	0.774**	0.0001		0.710**	0.0001	
LDL/HDL	0.586**	0.0001		0.510**	0.0001	

** Stands for *P <* 0.01. *R* Pearson’s correlation coefficient, *TG* Triglyceride, *TC* Total Cholesterol, *LDL* Low-density lipoprotein Lipoprotein, *HDL* High-density lipoprotein Lipoprotein, *TC/HDL* Total Cholesterol to High Density Lipoprotein Ratio, *LDL/HDL* Low-density lipoprotein to High-density lipoprotein ratio. Tests were anylysed using Pearson’s correlation and multiple regression test

## Discussion

As the present study showed, comparable lipid profile was seen among overweight/obese subjects which this finding was supported with former studies as well [31–33]. Based on the present knowledge depends on fat quality intake in obese and non-obese, chronic diseases such as hypertension and CVD could be prevented [34]. In the present study, participants with higher BMI had greater body fat percentage as well as AIP which was in line with the previous studies. Although it has shown that body fat distribution is associated with cardiometabolic risk factors [35], a recent study has shown that BMI by itself has a superior correlation with cardiovascular disease risk as compared to body fat [36]. Moreover, there is a positive correlation between BMI and lipid abnormalities, including increased TC, LDL cholesterol, and TG, which directly affect AIP as one of the most important risk factors for cardiovascular disease [37, 38]. In the present study, a positive correlation between AI, TI, SFA, MUFA, PUFA, and CSI with AIP and inverse association between h/H and AIP were noticed. These findings were similar to previous studies [39, 40]. Besides, likewise former findings all lipid profile and AIP showed a significantly positive correlation, while a significant negative correlation was found between HDL and AIP in both groups [41, 42]. Dietary fatty acids, determine the risk and protection of chronic diseases due to the chain length, number, and position of the double bonds as well as their shape (cis or trans) [43–45]. Therefore, fat quality intake can directly or indirectly have a profound effect on blood pressure and atherosclerosis [46, 47]. The main dietary fat risk factors for CHD which could be modified in food daily intake are fats high in TC and SFA, whereas USFA includes USFAs with multiple n-6 series (Linoleic), n-3 (Linolenic), a double bond USFA, have benefits in human life [23]. Diet recommendations and policies, largely based on previous studies, have shown that reducing SFA intake can prevent chronic diseases [48]. Evidence suggests that increasing the SFA diet, most importantly Lauric, Myristic, Stearic and Palmitate acid, increases LDL lipoprotein, and eventually being a serious risk factor for CHD [49, 50]. AI and TI are two other indicators that indicate the potential for stimulating platelet aggregation. According to the findings of Ulbricht, atherogenic proteins bind lipids to the immune and circulatory cells, and anti-atherogenic prevent plaque accumulation and reduce levels of fatty acid steroids, cholesterol, and phospholipids, prevent the development of micro and macro CHD and TI indicate a tendency to form clots in the blood vessels [23]. Foods with low levels of AI and TI (less SFA) have a greater potential for protection against coronary artery disease. Furthermore, the lipid profile associated with high trans fatty acids and palmitic acid and low consumption of linoleic acid has been associated with an increased risk of CVD [51]. Although, there is evidence that higher consumption of MUFA (consists of oleic acid) improves risk factors for CVD. The study showed that there was an inverse association between USFA intake and the risk of CVD, a meta-analysis of long term studies (more than 6 months) comparing the consumption of high MUFA diets to low MUFA diets found that high MUFA diets were associated with lower fat mass, and lower systolic and diastolic blood pressure [52]. In this regard, the major types of PUFA include ɷ-6 and ɷ-3 that some prospective cohort studies have shown that PUFA increases the risk of cardiovascular outcomes [53]. Although, prospective studies and randomized controlled trials provide strong evidence that replacing dietary SFA with USFA, both MUFA and PUFA, benefit cardiovascular health [50]. Another h/H as another indicator Ratio is an important additional index to determine the effect of individual fatty acids on cholesterol metabolism [24]. In terms of the nutritional value, a greater h/H ratio is directly proportional to a high PUFA content, which is considered more beneficial for human health. The low CSI index indicates a decrease in SFA and cholesterol and ultimately a decrease in atherogenicity. CSI may be used to compare different foods and recipes and to quickly and easily evaluate daily intake [25]. All of the above corresponds to our findings. The present study failed to find significant differences between normal and overweight/obese subjects in terms of the type of fatty acid intake. This failure might be due to either close gap of BMI between these studied subject groups, or similarly of the collected subjects in other variables like education, economic status, and so on. It might be possible to face significant results if only the obese subjects were included in the present study.

Many researchers believe that a ratio of ɷ-6/ɷ-3 in the human diet should not exceed 0.5 [54]. The benefits of ω3 PUFAs for humans are associated with the synthesis of eicosanoids, such as leukotrienes, prostaglandins, and thromboxanes [55–57]. Moreover, the indices of dietary fat quality were originally developed to investigate CVD and had not been previously tested for blood biomarkers in obese and non-obese [23]. Finally, this study showed that the type of dietary fat quality and the increase in obesity and overweight caused an increase in AIP, which was significantly correlated positively with lipid profiles and the atherogenic index of food. Several cross-sectional studies have shown that chronic diseases such as hypertension, dyslipidemia, and CVD are more prevalent in obese individuals [58]. According to the findings of the previous studies, there is a direct relationship between an increase in the blood atherogenic index and obesity, which ultimately predicts CVD [42]. Therefore, taking care of healthy food intake is an important factor to prevent chronic disease for all people.

### Study strengths and limitations

According to the searched data bank, this study was one of the pioneer studies in this field which has focused on this area. This study, however, has a few inherent limitations. First, it was a cross-sectional study with quite moderate in size. Secondly, a wide range of ages was included in this study which might have introduced a bias for the results. Therefore to have a better conclusion larger-scale studies with different age groups could show a better and clearer view of findings. Moreover, to have a more concrete conclusion it is highly recommended to do a cohort study in the future.

## Conclusion

In summary, the findings of the present study suggest a direct relationship between dietary fat quality, increased BMI, and abnormalities of lipid metabolism with AIP, which could ultimately be used as a contributing factor to CVD prediction. Even though further studies are needed to have tangible recommendations, meanwhile, taking care of dietary fat quality among people to prevent CVD would be a wise decision.

## Data Availability

Data supporting the results of this study are available from the Islamic Azad University’s Science and Research Branch (SRBIAU) clinic, but limitations apply to the use of these data, which have been used under license for the current analysis and are therefore not accessible to the public. However, data are available from the writers with the permission of the clinic and upon fair request. It has been stated in our contract between the clinic and us that they never send us details about the participants because our data are part of a great database. Even they have their own competent statistics expert who analyzes our findings, the results were written based on his report.

## Abbreviations

AIP: Atherogenic Index of Plasma
AI: Atherogenicity Index
TI: Thrombogenicity Index
h/H: Hypo/hypercholesterolemic ratio
CSI: Cholesterol-Saturated Fat Index
NCDs: Non communicable diseases
SBP: Systolic blood pressure
DBP: Diastolic blood pressure
CHD: Coronary heart diseases
CVD: Cardiovascular disease
BMI: Body mass index
SMM: Skeletal muscle mass
FFQ: Food frequency questionnaire
LDL-C: Low-density lipoprotein cholesterol
HDL-C: High-density lipoprotein cholesterol
TG: Trigliceride
TC: Total cholesterol
MUFA: Monounsaturated fatty acids
PUFA: Poly-unsaturated fatty acid
SFA: Saturated Fatty acids
USFA: Unsaturated Fatty acids
